# A brain extraction algorithm for infant T2 weighted magnetic resonance images based on fuzzy c-means thresholding

**DOI:** 10.1038/s41598-021-02722-0

**Published:** 2021-12-02

**Authors:** Inyoung Bae, Jong-Hee Chae, Yeji Han

**Affiliations:** 1 Department of Health Sciences and Technology, Gachon Advanced Institute for Health Sciences and Technology, Gachon University, Incheon, Republic of Korea; 2 Department of Pediatrics, Seoul National University College of Medicine, Seoul, Republic of Korea; 3 Department of Biomedical Engineering, College of Health Sciences, Gachon University, Incheon, Republic of Korea

**Keywords:** Anatomy, Mathematics and computing

## Abstract

It is challenging to extract the brain region from T2-weighted magnetic resonance infant brain images because conventional brain segmentation algorithms are generally optimized for adult brain images, which have different spatial resolution, dynamic changes of imaging intensity, brain size and shape from infant brain images. In this study, we propose a brain extraction algorithm for infant T2-weighted images. The proposed method utilizes histogram partitioning to separate brain regions from the background image. Then, fuzzy c-means thresholding is performed to obtain a rough brain mask for each image slice, followed by refinement steps. For slices that contain eye regions, an additional eye removal algorithm is proposed to eliminate eyes from the brain mask. By using the proposed method, accurate masks for infant T2-weighted brain images can be generated. For validation, we applied the proposed algorithm and conventional methods to T2 infant images (0–24 months of age) acquired with 2D and 3D sequences at 3T MRI. The Dice coefficients and Precision scores, which were calculated as quantitative measures, showed the highest values for the proposed method as follows: For images acquired with a 2D imaging sequence, the average Dice coefficients were 0.9650 ± 0.006 for the proposed method, 0.9262 ± 0.006 for iBEAT, and 0.9490 ± 0.006 for BET. For the data acquired with a 3D imaging sequence, the average Dice coefficient was 0.9746 ± 0.008 for the proposed method, 0.9448 ± 0.004 for iBEAT, and 0.9622 ± 0.01 for BET. The average Precision was 0.9638 ± 0.009 and 0.9565 ± 0.016 for the proposed method, 0.8981 ± 0.01 and 0.8968 ± 0.008 for iBEAT, and 0.9346 ± 0.014 and 0.9282 ± 0.019 for BET for images acquired with 2D and 3D imaging sequences, respectively, demonstrating that the proposed method could be efficiently used for brain extraction in T2-weighted infant images.

## Introduction

To obtain useful clinical information from the brain, magnetic resonance (MR) images undergo several image-processing steps, such as registration, filtering, and segmentation. While various image analysis techniques are utilized to extract valuable information from the MR images, analyzing the volumetric information of brain structures using T2-weighted (T2w) MR images is essential in making an accurate diagnosis of infant brain diseases. For example, T2w MR images can provide CSF volume measurements, which are utilized for evaluation of brain atrophy, and other sub-volume measurements, which can be utilized as anatomical and pathological information^1,2^. In the case of an infant's brain disease, abnormal T2 intensities can provide crucial features for diagnosis; for example, the periventricular and deep white matter shown in T2-weighted images can be a clue to identifying diseases such as periventricular leukomalacia and metachromatic leukodystrophy^3,4^. To obtain such brain features, skull stripping, i.e., isolating the brain regions from non-brain regions in an image, is an essential image pre-processing procedure for MR images and the results of skull stripping may affect the accuracy of the deduced brain features. This is especially true in an infant's brain where the brain growth and developmental changes can be observed based on the brain volume acquired by the skull stripping algorithms^5^.

Frequently used methods for skull stripping include the brain extraction tool (BET), statistical parameter mapping (SPM), brain surface extractor (BSE), and model-based level sets (MLS), which are useful for application in adult T1-weighted (T1w) images^6–9^. Since an adult's brain MR image has more distinct contrast than an infant’s brain MR image and T1w images are primarily used as anatomical images for adult brains, most of the conventional skull stripping algorithms are developed and optimized for adult T1w images. Infant MR images are considerably more challenging for skull stripping, due to their low spatial resolution, dynamic changes of imaging intensity, brain size and shape^10^, and the algorithms developed for adult brains are often not suitable for infant brains^11,12^. Apart from the size and shape, the most significant differences in adult and infant brains are as follows; Infant brains between 0 and 6 months present reverse contrast with adult brains (In T2w image, white matter (WM) intensity is higher than gray matter (GM) intensity). Infant brains between 8 and 12 months show poor contrast between WM and GM, and the early-adult pattern represents over 12 months infant image^13^. For the above-mentioned reasons, brain anatomy is revealed more clearly in T2w than in T1w images in neonate and early infant brain images and T2w images are considered the primary images for anatomical segmentation^14^.

Although it is important to process T2w infant brain images, the conventional skull stripping methods may not generate the best results for infant brain images because it is more difficult to distinguish the brain from non-brain regions such as skull, dura, and muscles with the same algorithms used for adult^15,16^. To this end, only a few methods have been developed for skull stripping of infant brain MR images^10^. For example, BET was optimized for both T1- and T2-weighted brain images and applied to adults’ and infants’ brain images^14^. However, the brain extraction results of infant images were relatively less accurate than those of adult brain images and the results highly depended on the used parameters^6^. Recently, deep-learning based approaches have been also introduced for infant brain extraction. One of the most frequently used tools is a learning-based method which combines a meta-algorithm and level-set fusion, called the Infant Brain Extraction and Analysis Toolbox (iBEAT)^17^ and it was optimized for both T1- and T2-weighted infant brain images. U-net, which is a commonly used architecture for deep learning-based medical image segmentation^18^, can be also applied to infant brain extraction. In addition, a pre-trained deep learning brain extraction method (PARIETAL) performed successfully with T1-weighted adult brains on three different scanners^19^ and the convolutional neural network (CNN) was also successfully adopted to extract the brain regions, trained by T1-wighted healthy or diseased adult images, and the fetal T2-weighted images scanned at a gestational age between 19 and 39 weeks^20^. However, the segmentation performance of the deep-learning based approaches largely depend on the characteristics of the training and target datasets, which is limitation of many machine-learning based image processing techniques^18,21,22^.

Due to above-mentioned reasons, obtaining accurate brain masks in infant’s brain T2-weighted images is not straightforward and popular methods do not produce satisfactory results for neonatal images^23^. Thus, we have developed an efficient brain extraction algorithm for infant T2-weighted images to work around the difficulties of the existing methods and performed experiments with normal to slightly abnormal datasets (ages 0–24 months). Since the proposed method is optimized for infant T2-weighted brain images, it is particularly helpful to perform accurate infant brain extraction for research and clinical purposes. In the following sections, the basic principle of the proposed skull stripping approaches is outlined and the experiment results are presented with special emphasis on performance, sensitivity to image artifacts, and potential applications.

## Materials and methods

Before applying the algorithm, it is necessary to standardize the brain volume. Thus, the input images were reoriented with the RAS (right, anterior, and superior) coordinates^7^ and the N3 bias correction was performed to remove image inhomogeneity^24^. Then, de-noising was performed using low pass filtering (LPF) and anisotropic diffusion filtering (ADF)^25^.

After pre-processing, the proposed algorithm takes a slice-by-slice approach in axial slices to extract brain regions; we initially start the skull-stripping at the center slice and then move towards the upper and lower slices. In each slice, the proposed method obtains a rough brain mask through fuzzy c-means (FCM) thresholding^26^. Using the rough brain mask and spatial information of the neighboring slices, a refined brain mask is generated. Finally, a step to remove the eye is additionally proposed for slices that include eyes. More details of the algorithm are explained in the following sections.

### Background removal

As the first step to acquire a rough brain mask, the proposed method separated the brain regions from the background in the axial slices using the image histogram^27^. In an MR image histogram, the object image can be roughly distinguished from the background because they are represented as high peaks in the clusters of the intensities. Based on the histogram partitioning, concavities around the main peaks of a histogram can be detected, which can be used to separate the brain region from non-brain regions as follows.

**Figure 1:**
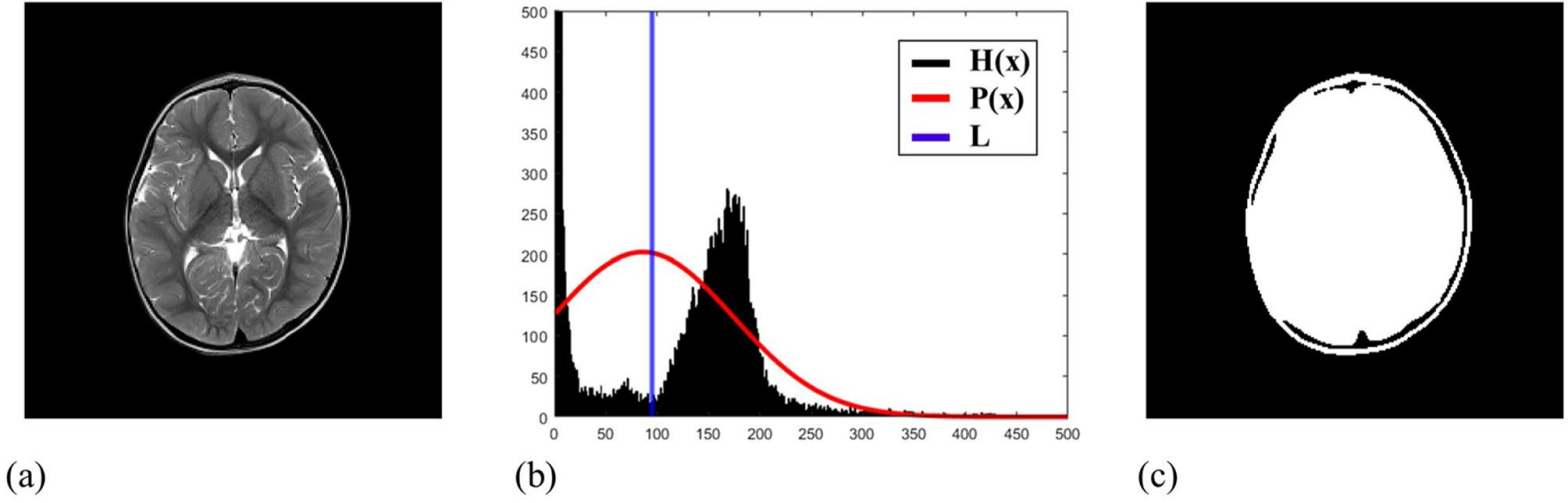
The process of histogram partitioning. (**a**) The original MRI brain image. (**b**) The histogram of the MR image is utilized to divide the background and the brain regions. H(*x*) is the histogram of the MR image and P(*x*) is the normal distribution graph having the same mean and standard deviation as H(*x*). The blue line (L) represents the calculated threshold for background removal. (**c**) The final result of histogram partitioning.

To partition the histogram *H*(*x*) of an MR image, we use a Gaussian graph *P*(*x*), defined on the same gray level as *H*(*x*) (Fig. 1). Specifically, *P*(*x*) is defined as a normal distribution, having the identical mean (*μ*) and standard deviation (*α*) as *H*(*x*), so that the areas under the graphs are identical for *P*(*x*) and *H*(*x*). Thus, *P*(*x*) can be defined as,

1 $$ {\text{P}}\left( x \right) = \alpha /z*G\left( x \right), $$

where *G*(*x*) is the probability density function with the standard normal distribution ($\mu = 0, \alpha = 1$)and *P*(*x*) has the highest peak at *x* = *μ*. *z* is the summation of *G*(*x*) for $x_{\min } < x < x_{\max }$ ($x_{\min } = 0$ and $x_{\max }$ is the highest intensity value in the histogram). In *H*(*x*), it can be assumed that the highest intensity peaks are also located around *x* = *μ* because the large number of pixels have large influence on the mean value and the concavities surrounding these peaks are often in contrast with the convex part of *P*(*x*)^27^. Therefore, we find the intensity that has the maximum divergence between *P*(*x*) and *H*(*x*) near the mean value, as described in Eq. (2).

2 $$ {\text{L}} = \begin{array}{*{20}c} {\arg max} \\ x \\ \end{array} \left( {P\left( x \right) - H\left( x \right)} \right), $$

where *L* is the intensity value of the estimated concave valley, which can be used as the threshold value to obtain the background-removed binary images, as shown in Fig. 1c.

### Obtaining the rough brain mask

As the intensities near the skull area vary, it is hard to separate uncertain tissues, including the dura and muscles, from brain tissues using a single threshold. Thus, we adopted the fuzzy c-means clustering method, which performs classification by maximizing the non-similarity in inter-cluster datasets and similarity in intra-cluster elements by iterative calculations. As the FCM clustering allocates the data based on the probability or membership values^28^, it is appropriate for classification of image pixels with ambiguous intensity differences.

To apply the FCM clustering, the number of clusters (*c*) needs to be selected a priori. In this work, *c* was experimentally selected to distinguish the brain tissues from the rest. The procedure of selecting *c* is explained in “Parameter settings” section. The FCM algorithm basically aims to minimize the sum-of-squares error (SSE) between the measured data $x_{i} = \left\{ {x_{1} \ldots x_{n} } \right\}$ and the centroid of the cluster $c_{j} = \left\{ {c_{1} \ldots c_{c} } \right\}$, as follows.

3 $$ \min SSE = \mathop \sum \limits_{i = 1}^{n} \mathop \sum \limits_{j = 1}^{c} \omega_{ij}^{p} d\left( {x_{i} , c_{j} } \right)^{2} $$

In Eq. (3), $\omega_{ij}$ denotes the degree of membership of *x*_*i*_ in the cluster *j*, defined as follows.

4 $$\upomega _{{{\text{ij}}}} = 1/\mathop \sum \limits_{k = 1}^{c} \left\{ {d\left( {x_{i} ,c_{j} } \right)^{2} /d\left( {x_{i} ,c_{k} } \right)^{2} } \right\}^{2/p - 1}, $$

where *d*(*x*_i_, *c*_j_) is the metric distance between the element *x*_i_ and the centroid *c*_j_, which is calculated as

5 $$ {\text{c}}_{{\text{j}}} = \mathop \sum \limits_{i = 1}^{n} \omega_{ij}^{p} x_{i} /\mathop \sum \limits_{i = 1}^{n} \omega_{ij}^{p} $$

*p* is a real number greater than 1 and 2 is normally used to simplify the calculation^28^. In short, the membership value $\omega_{ij}$ represents the probability of element $x_{i}$ belonging to the cluster *j*.

Once the centroid of each cluster is updated, the membership value is calculated again. The iteration process of calculating the SSE is repeated until the local extreme of the objective function (Eq. 3) provides an optimal clustering, which has the maximum similarity within the clusters based on the following criterion.

6 $$ \min \;SSE < \varepsilon ,\quad {\text{then}}\;STOP $$

In this work, the optimal termination threshold (*ε*) of 0.02 was experimentally selected (“Parameter settings” section).

After conducting the FCM clustering, each pixel is assigned to the cluster with the maximum membership value. Then, we can calculate the threshold values (*th*_m_) that separate *m*_th_ and (*m* + 1)_th_ clusters by averaging the maximum intensity of the *m*_*th*_ cluster (*max*(*I*_*m*_)) and the minimum intensity of the (*m* + 1)_th_ cluster (*min*(*I*_*m*+*1*_)) as,

7 $$ th_{m} = \left( {\max \left( {I_{m} } \right) + \min \left( {I_{m + 1} } \right)} \right)/2,\quad for\quad m = 1 \ldots \left( {c - 1} \right). $$

By using the generated (*c* – 1) thresholds, different brain masks (mask #*m*) can be generated for each slice. In this work, mask #2 was selected as the rough brain mask, which initially outlines the brain region, for brain extraction after experimentally assessing masks for *m* = 1…7. For detailed explanation of the assessment, please read “Parameter settings” section.

### Refinement

**Figure 2:**
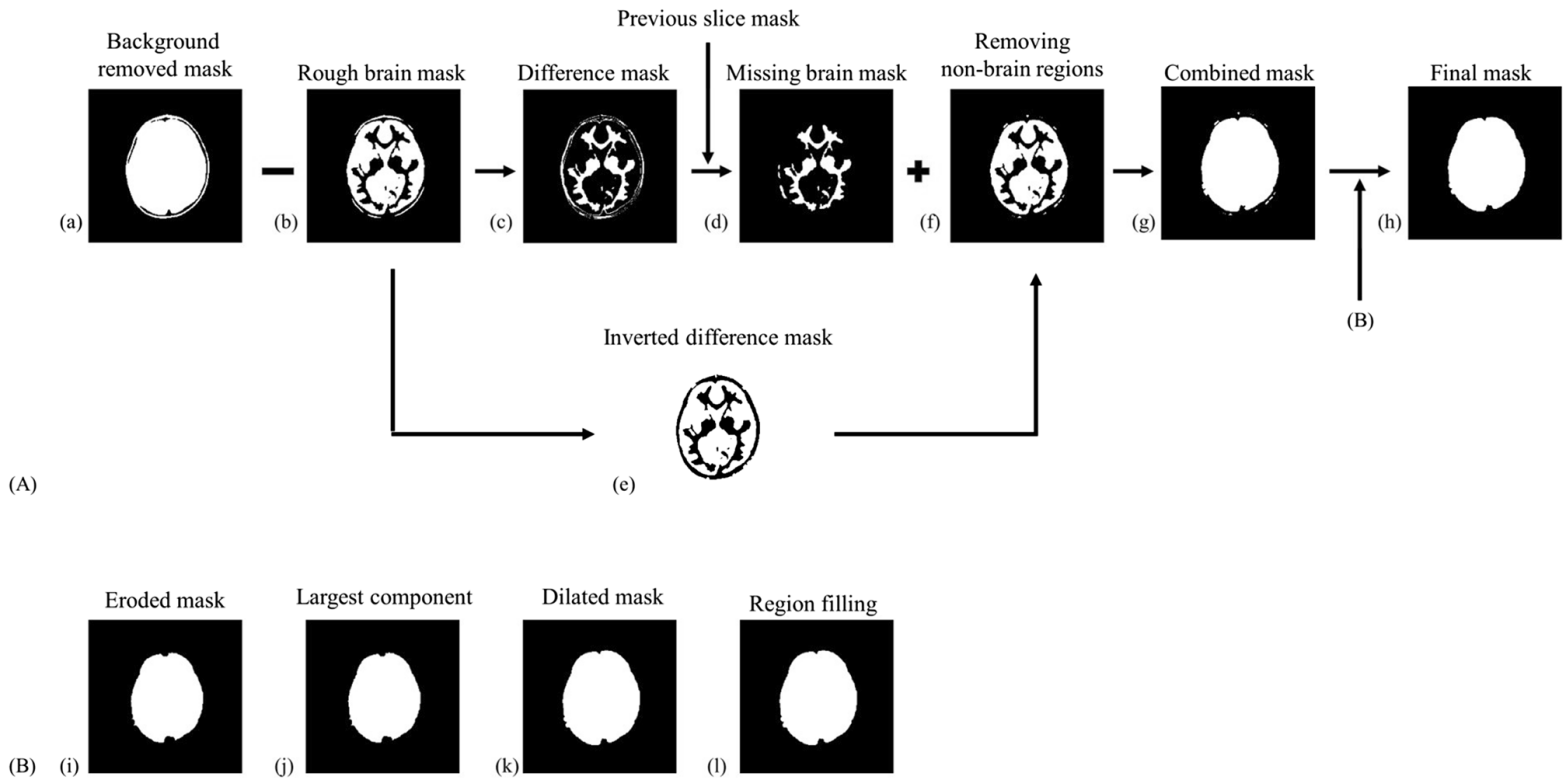
The overall process of the proposed brain extraction algorithm. (**A**) (**a**) The background removal result of histogram partitioning, (**b**) the rough brain mask after fuzzy c-means thresholding, (**c**) the difference mask of the background-removed mask and the rough brain mask ((**a**)-(**b**)), (**d**) the missing brain regions ((**c**) ∩ previous slice mask ((**i**) of the previous slice)), (**e**) the inverted difference mask, which is used to remove the non-brain regions from the rough mask, (**f**) the brain mask after elimination of non-brain regions ((**b**) ∩ (**e**)), (**g**) the combined brain mask ((**d**) + (**f**)), and (**h**) the final brain mask after the morphological operations (**B**).

The refinement process aimed to fine-tune the rough brain mask using the spatial information of the neighboring slices. Specifically, the spatial information of the previous slice was used to remove the non-brain region in the current image slice (Fig. 2) because the proposed brain extraction algorithm was sequentially applied from the center slice to the outer slices.

In this step, the rough brain mask generated in “Obtaining the rough brain mask” section (Fig. 2b) was subtracted from the background-removed brain mask (Fig. 2a) to generate a difference mask that initially estimated which brain regions might be missing in the rough brain mask, as shown in Fig. 2c. Then, the overlapping regions between the difference mask (Fig. 2c) and the brain mask of the previous slice were acquired to estimate the missing brain regions in the rough mask (Fig. 2d). However, the estimated missing brain regions (Fig. 2d) could also include some non-brain regions. To eliminate the non-brain regions, the overlapping regions between the inverted difference mask (Fig. 2e) and the rough mask (Fig. 2b) was additionally obtained (Fig. 2f). Then, a combined mask (Fig. 2g) can be obtained by adding the brain mask of the estimated missing brain regions (Fig. 2d) and the overlapped regions (Fig. 2f). Finally, morphological operations (erosion, selecting the largest connected component, dilation, and region filling)^29^ were performed to the combined brain mask (Fig. 2g). Consequently, we can obtain the final mask (Fig. 2h). As an exception, the middle slice of the whole volume does not have a previous slice because it is the first slice to be tackled. Therefore, only the morphological operations (Fig. 2B) were applied to the rough brain mask of the middle slice with a 3 × 3 spherical structuring element as refinement.

### Removing the eyes

In certain slices that include the eyeball regions, the refined brain mask would still have errors around the eyeballs. Thus, the slices with eyeballs are automatically selected to eliminate the non-brain tissues, as follows.

**Figure 3:**
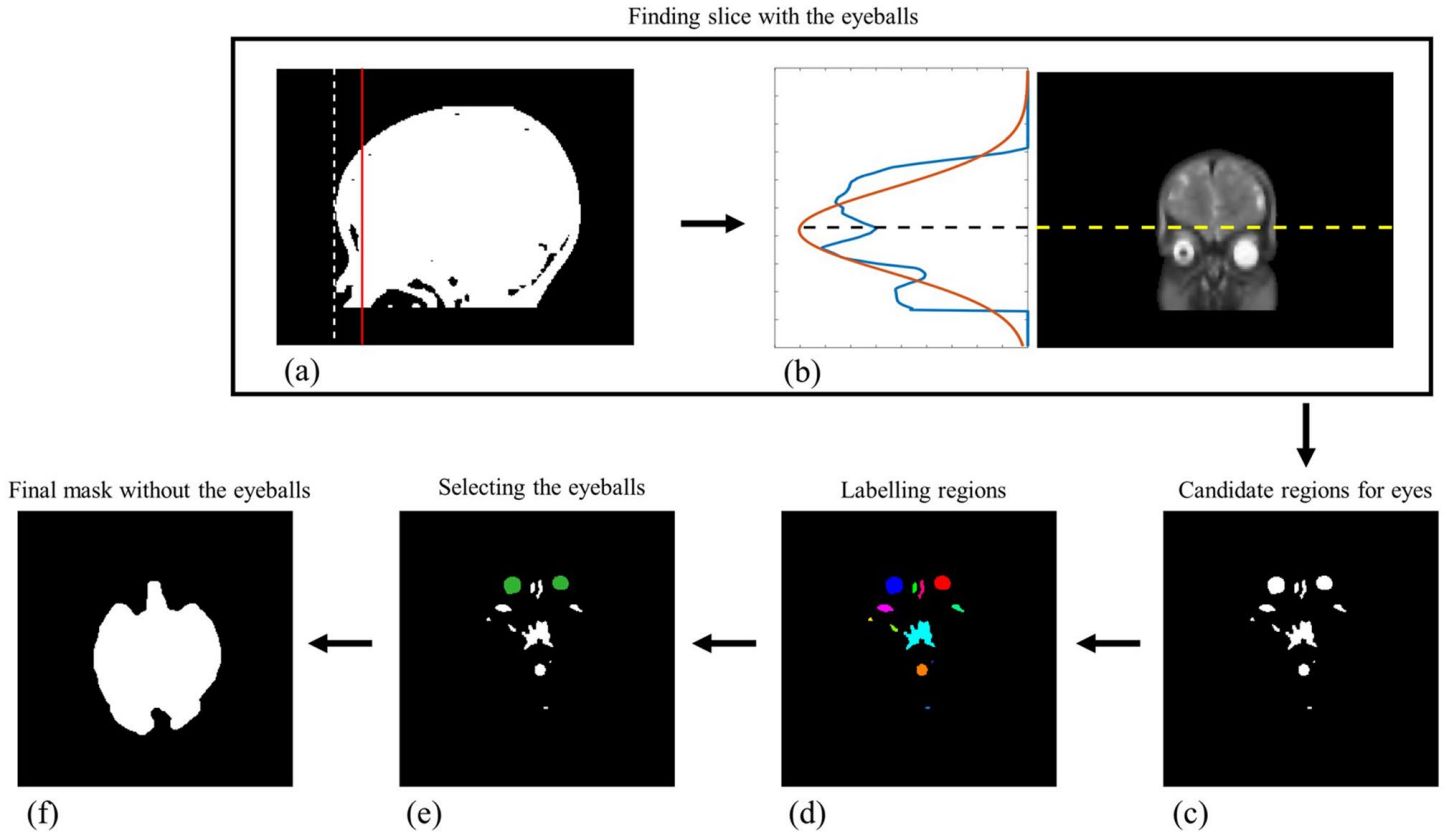
The proposed eye removal algorithm. (**a**) In the sagittal slice with the longest anterior–posterior distance, a coronal slice that includes the vertical line (red) at a 15 mm distance from the front-most vertical line (dotted white line) is selected. (**b**) In the coronal slice, the starting position of the eyes is detected. (**c**) Candidate regions for eyes. (**d**) Labelling the candidate regions. (**e**) Selecting the eyeball regions. (**f**) The final brain mask after eliminating the eyeball regions.

As the first step to locate the eye area, a sagittal image slice with the longest anterior–posterior distance was selected from the sagittal image slices. In that sagittal slice, we placed a vertical line at a 15 mm distance from the front-most vertical line (Fig. 3a), considering the thickness of the skull and the average diameter of the infant's eyes^30–32^. By taking the coronal slice that contained the vertical line, the coronal slice with the eyeballs could be selected, where the projection in the left–right direction showed a peak near the eye position (Fig. 3b), because the eyes were represented as high intensities. Then, the maximum divergence between the projection graph and the Gaussian graph was found to designate the starting position of the axial slices (black line) that include the eyes (Fig. 3b), and the following eye-removal algorithm is applied to the slices starting from the starting position.

Using a pre-defined threshold (“Parameter settings” section), candidate eye regions in an axial slice could be selected (Fig. 3c) and different connected regions were labeled (Fig. 3d) using the connected-component labeling algorithm^29^. Then, the candidates for eye regions were reduced from the labeled regions based on the features of the eyes; Since the eyes are located in the anterior region of the brain, only the candidates in the front half area of the axial plane are considered. Additionally, the shape similarity between the candidate regions and a circular model was calculated using the bidirectional Hausdorff distance^33^, as follows,

8 $$ {\text{HD}}\left( {{\text{A}},{\text{B}}} \right) = {\text{max}}\left\{ {{\text{h}}\left( {{\text{a}},{\text{b}}} \right),{\text{h}}\left( {{\text{b}},{\text{a}}} \right)} \right\}, $$

where *h*(*a, b*) denotes one-sided Hausdorff distance from *A* to *B* point sets, where $A = \left\{ {a_{1} ,a_{2} , \ldots ,a_{n} } \right\}$ *and* $B = \left\{ {b_{1} ,b_{2} , \ldots ,b_{m} } \right\}$, defined as *h*(*A,B*) = *d*(*a*, *b*). Thus, a shorter Hausdorff distance represents higher similarity with each point set. In this work, a circular model with a 20 mm diameter was used for the reference point set by considering the average diameter of infant's eyes. By analyzing the shape similarity with the circular model, the eye regions could be determined from the candidates (Fig. 3e). The final brain mask without the eye regions could be obtained by eliminating the estimated eye regions from the brain mask (Fig. 3f).

### Approval for human experiments

Informed consents were obtained from all participants and/or their legal guardians. This study was approved by the Seoul National University Hospital Institutional Review Board (IRB no: 1607-059-755) and was performed in accordance with the relevant guidelines and regulations.

## Results

### Data preparation

To validate the proposed algorithm, we used T2 infant brain images, which were previously acquired with a 2D Turbo Spin Echo (TSE) sequence and a 3D TSE BLADE sequence. T2 infant images from 2D TSE sequence are aged 7–24 months (mean ± STD = 11.77 ± 5.16, 9 subjects) and infant T2-weighted images of 3D TSE BLADE are aged 2–24 months (mean ± STD = 13.81 ± 7.73, 16 subjects), acquired at 3T MRI from different vendors (Philips, Siemens). The TSE datasets contained infant brain images with no structural abnormalities and the TSE BLADE datasets contained images from normal infants and infants with pathological characteristics, including cystic lesions, hypo- and hyper-intensities. Institutional review board approval was acquired before processing the data using the proposed method.

For validation, we also used the dHCP (developing Human Connectome Project) dataset^14^, which provide images of neonatal brains with ages of 37–44 gestational weeks. In dHCP dataset, the T2w MR data and the brain extraction masks acquired with BET are provided for public use. However, it should be noted that the dHCP dataset was provided after “defacing” and thus it is not suitable for our study because the proposed method is targeted for processing the MRI data in its original rawdata format. Nevertheless, we used the first 50 datasets of the dHCP to demonstrate the feasibility of the proposed method because no other infant data was available.

In this work, the input images were prepared to have 1 × 1 × 1 (mm^3^) resolution with a matrix size of 256 × 256 × 198 after reorientation and resampling. For LPF, a kernel with a size of 3 × 3 was used to prevent removal of excessive structural details. For ADF, local gradient between the intensities of pixels was utilized to prevent the regions having rapid gradient changes from blurring, where the degree of blurring was controlled by the iteration and the diffusion constants^25^. For evaluation of the proposed brain extraction algorithm, manually segmented brain images were also prepared with reference to^34^ by neurology experts.

### Qualitative measures

After performing brain extraction using the proposed method, iBEAT, and BET, the results were compared with the manual segmentation results using Dice coefficient and Precision measures^35,36^. Dice coefficient is the most commonly used measure for shape similarity, which describes how well the result image fits in the reference image. Precision shows the percentage of true brain region within the detected regions. The accuracy of the segmentation results could be analyzed by comparing the Dice coefficients of the manually segmented result (A) and the brain masks (B) generated by different algorithms. The Dice coefficient represents the similarity between the two objects as

9 $$ \textit{Dice\,coefficient} = 2\left| {AB} \right|/(\left| A \right| + \left| B \right|). $$

Precision can be also calculated to assess the fraction of true detection within the whole detections of an algorithm as

10 $$ \textit{Precision} = (\textit{True\,detection})/(\textit{Whole\,detections\,of\,an\,algorithm}). $$

In this work, true detection was determined by overlaying the brain extraction results with the manually segmented images.

### Parameter settings

To apply the proposed brain extraction algorithm, the parameters of the fuzzy c-means algorithm, i.e., the number of clusters and the terminal threshold for iteration, must be decided first so that the rough brain mask can be obtained. Thus, we evaluated the objective function with respect to different numbers of clusters and termination values.

**Figure 4:**
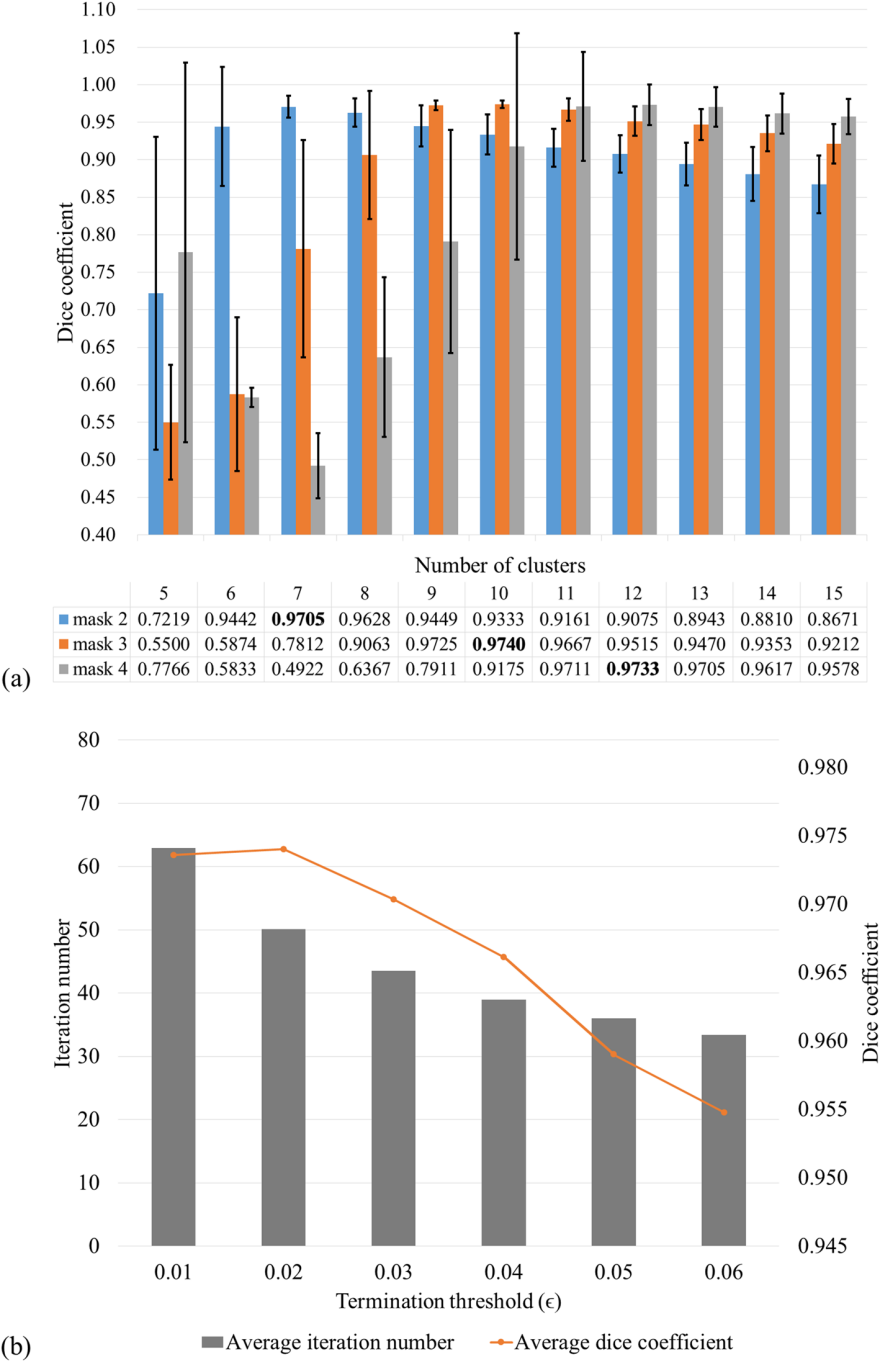
The optimal parameter setting. (**a**) Dice coefficient vs. mask #*m* (*m* = 2, 3, and 4) for different number of clusters (*c* = 5–15) are presented in this figure. (**b**) Iteration number vs. termination threshold. Different FCM thresholding values were utilized to find an optimal rough mask and an optimal termination threshold of 0.02 was selected based on the accuracy and computation time.

First, we evaluated the brain extraction results with respect to different numbers of clusters using a sufficient iteration number of 100 (Fig. 4a) to find an optimal *c*. By comparing the similarity between the manual segmentation and the final brain mask obtained by the proposed method using various combination of *c* and *m*, an optimal number of clusters was selected. In other words, we analyzed the performance of the proposed algorithm when brain mask #2, #3, or #4 (*m* = 2, 3, and 4) obtained from different numbers of clusters (*c* = 5–15) was used as the rough brain mask. Mask #1 and masks #5–14 were not evaluated because mask #1 included excessive non-brain regions and masks #5–14 did not include enough brain regions*.* The Dice coefficient was calculated for the brain masks generated with different parameters and the manually segmented image. According to Fig. 4a, mask #3 from 10 clusters generated the highest Dice coefficient; however, the differences with the brain masks generated with mask #2 from 7 clusters and mask #4 from 12 clusters were subtle. Consequently, we used mask #2 from 7 clusters for the rough brain mask in this study because it generally provided sufficient performance with the least amount of variance and processing time for different image data.

Then, we examined the termination threshold (*ε*) values ranging from 0.01 to 0.06 at an interval of 0.01 for brain extraction with mask #2 from 7 clusters, as determined in the previous step. Although 0.01 or less values were conventionally used for *ε*, it required a very long running time in this experiment. By comparing the average iteration numbers and Dice coefficients of the final mask, as illustrated in Fig. 4b, the termination threshold value of 0.02 was selected as for iteration to generate the highest similarity within reasonable running time.

For eyeball removal, we obtained the regions with higher intensities in each slice using a single threshold. In this work, we used the 60–100% range of the whole volume in the histogram to obtain a threshold value that could provide the brain regions of higher intensities, including CSF, fat, and the eyeballs. This is because the volume of white matter and gray matter represents almost 60% of the histogram in a one-month-old infant’s T2 weighted MR image, according to the previous research^37^.

### Comparison methods

To compare the performance of the proposed method with conventional methods, we used BET and iBEAT. For deep-learning based segmentation, we adopted the U-net architecture, which has a U-symmetric structure that includes encoding and decoding paths to apply pooling and upconvolution, respectively. The architecture is known to improve the network performance by integrating features from different levels while preserving the location information. In this work, the parameters of the U-net were given as follows: the number of channels in the first convolutional layer = 128, total depth = 5, and kernel size = 3. In each block, two sets of three layers, each of which consisted of a convolutional layer (or deconvolutional layer), ReLU layer, and a batch normalization layer, were used. To optimize the network, we used differentiable soft-Dice loss and ADAM optimizer with following parameters: base learning rate = $2 \times 10^{ - 5}$ and the number of epochs = 25. Weights of the network were initialized form Gaussian distribution with a zero mean and a SD of 0.001. The network and learning process were implemented environment with a TitanXP GPU processor.

To observe the performance of the U-net with respect to different characteristics of the datasets, we trained the network with three different dataset configurations: (i) 48 sets of dHCP images (9504 slices), (ii) 44 sets of dHCP + 4 sets from our dataset (9504 slices), and (iii) 25 sets from our infant data (4950 slices). Training the network with configurations (i) and (ii) required 4 h and training with configuration (iii) required 3 h. For each configuration, 2 dHCP datasets and 2 of our infant data were tested for validation. For label images, the brain masks provided by dHCP and manually segmented masks were used for dHCP and our infant data, respectively, and the data used for training were not used for testing.

After processing the data with different brain extraction methods, we compared the segmentation performance by calculating the Dice and Precision coefficients, where the manually segmented mask was used as the ground truth.

### Brain extraction results

**Figure 5:**
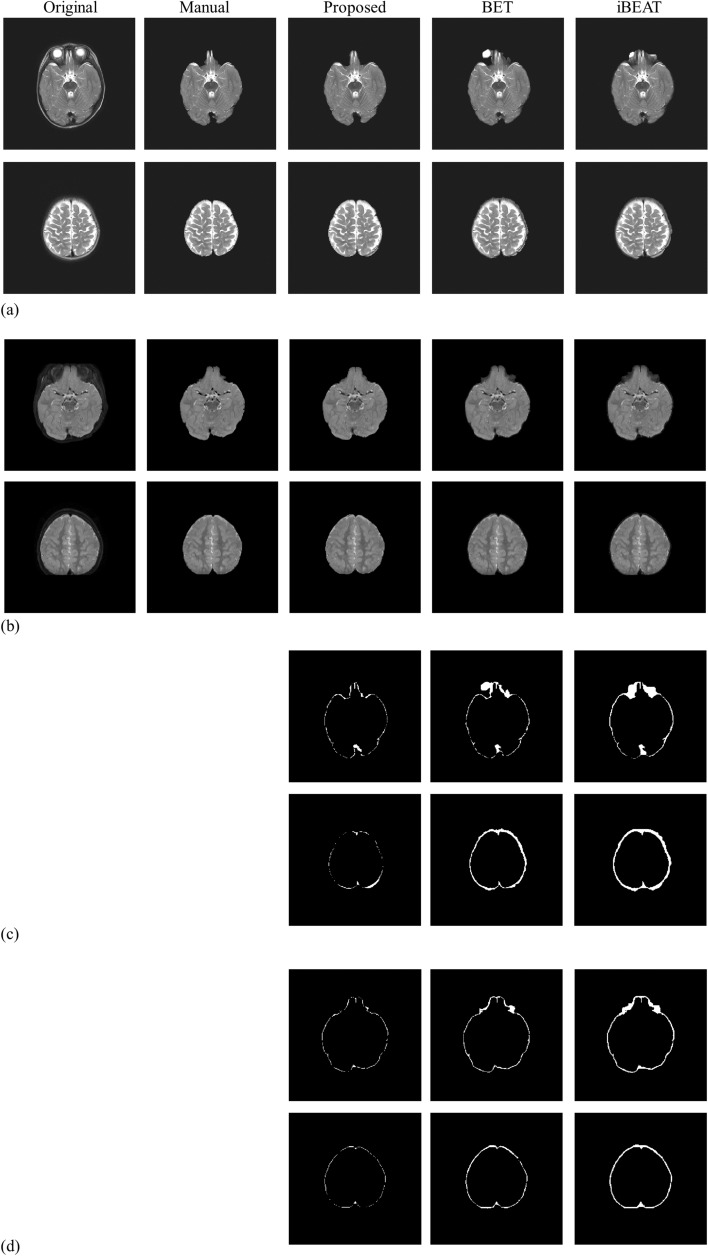
Representative images of brain extraction by the proposed method, BET, and iBEAT. (**a**) and (**b**) show selected slices from datasets acquired with 2D and 3D TSE sequences, respectively. (**c**) and (**d**) show difference images of the manual segmentation results and the corresponding brain extraction results of (**a**) and (**b**), respectively.

The brain extraction results generated by the proposed method and conventional methods are presented in Fig. 5, where Fig. 5a and b show selected slices from our infant datasets acquired with 2D and 3D TSE sequences, respectively. The images acquired with 2D and 3D sequences have different image contrasts due to sequence parameters and brain developmental stages of infants. In Figs. 5a and 5b, the leftmost two columns are the original images and the manually segmented images, followed by brain extraction images acquired by the proposed method, BET, and iBEAT. As demonstrated by the upper rows of Fig. 5a and b, the image slices including the eye regions are more challenging to separate brain and non-brain regions. However, the images demonstrated that the proposed method showed better brain extraction results even in the tricky regions. The difference images of the manual segmentation results and other brain extraction results are also presented in Fig. 5c and d. In general, the observed difference regions are reduced in the proposed method. The errors of BET and iBEAT are more apparent around the eye regions, where eye regions are not sufficiently removed in the BET images and muscles around the eyes are included in the iBEAT images. Regardless of different characteristics of images due to MRI sequences, the proposed method showed better performance than other algorithms.

**Figure 6:**
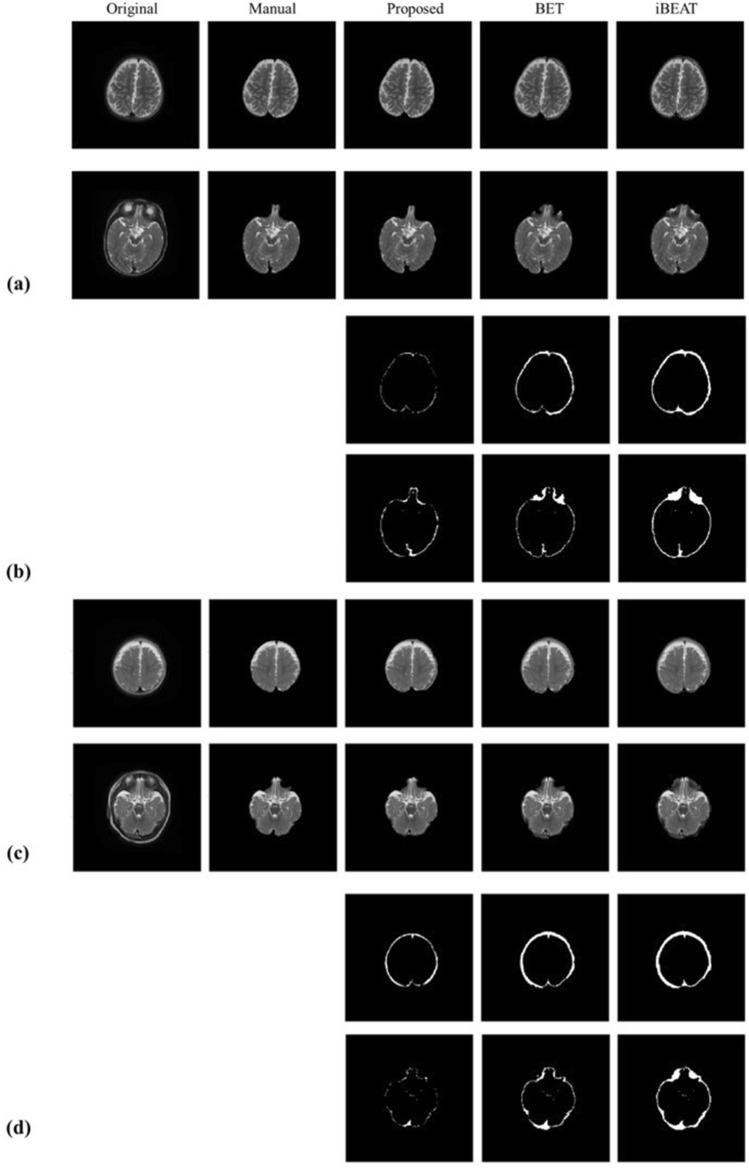
Brain extraction results of abnormal brain images generated by the proposed method, BET, and iBEAT. Images in (**a**) and (**c**) are selected from two different subjects. (**b**) and (**d**) show diffetence images of manual segmentation results and the corresponding brain extraction results of (**a**) and (**c**), respectively.

Selected slices from brain extraction results of abnormal brains are also provided in Fig. 6, where the subjects were reported to have a small choroid fissure cyst (a) and prominent subarachnoid CSF fluid with subdural fluid collection (c). In Fig. 6(b) and (d), the corresponding difference images are also presented. As demonstrated by Fig. 6, the proposed method can be applied to images with abnormal intensity changes to generate satisfactory brain extraction results.

**Figure 7:**
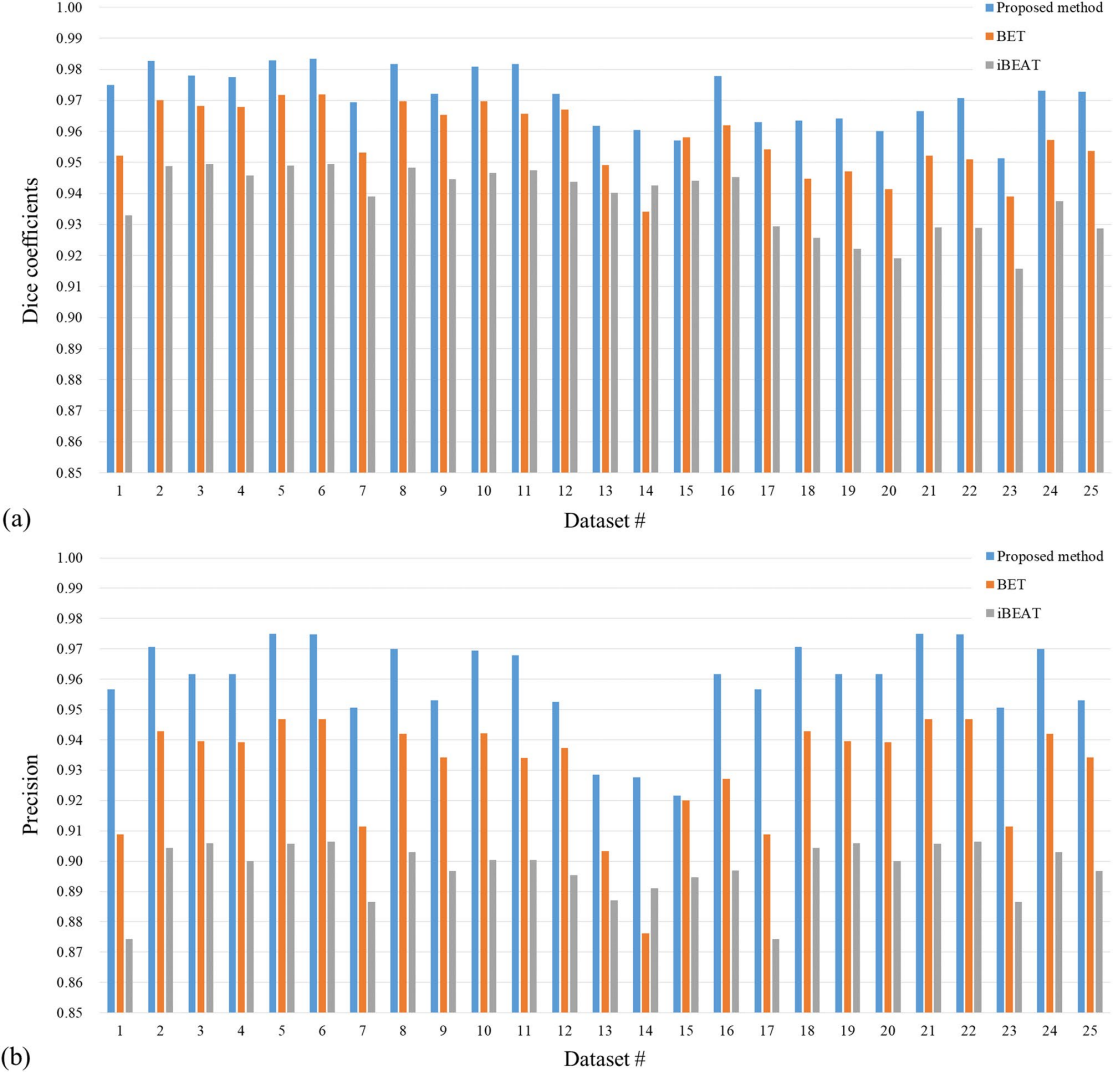
Quantitative analysis. Datasets #1–16 were acquired with a 3D TSE sequence and #17–25 were acquired with a 2D TSE sequence. (**a**) Dice coefficient graph. (**b**) Precision graph. The proposed method (blue) represents higher accuracy compared with the BET (orange) and iBEAT (gray).

**Figure 8:**
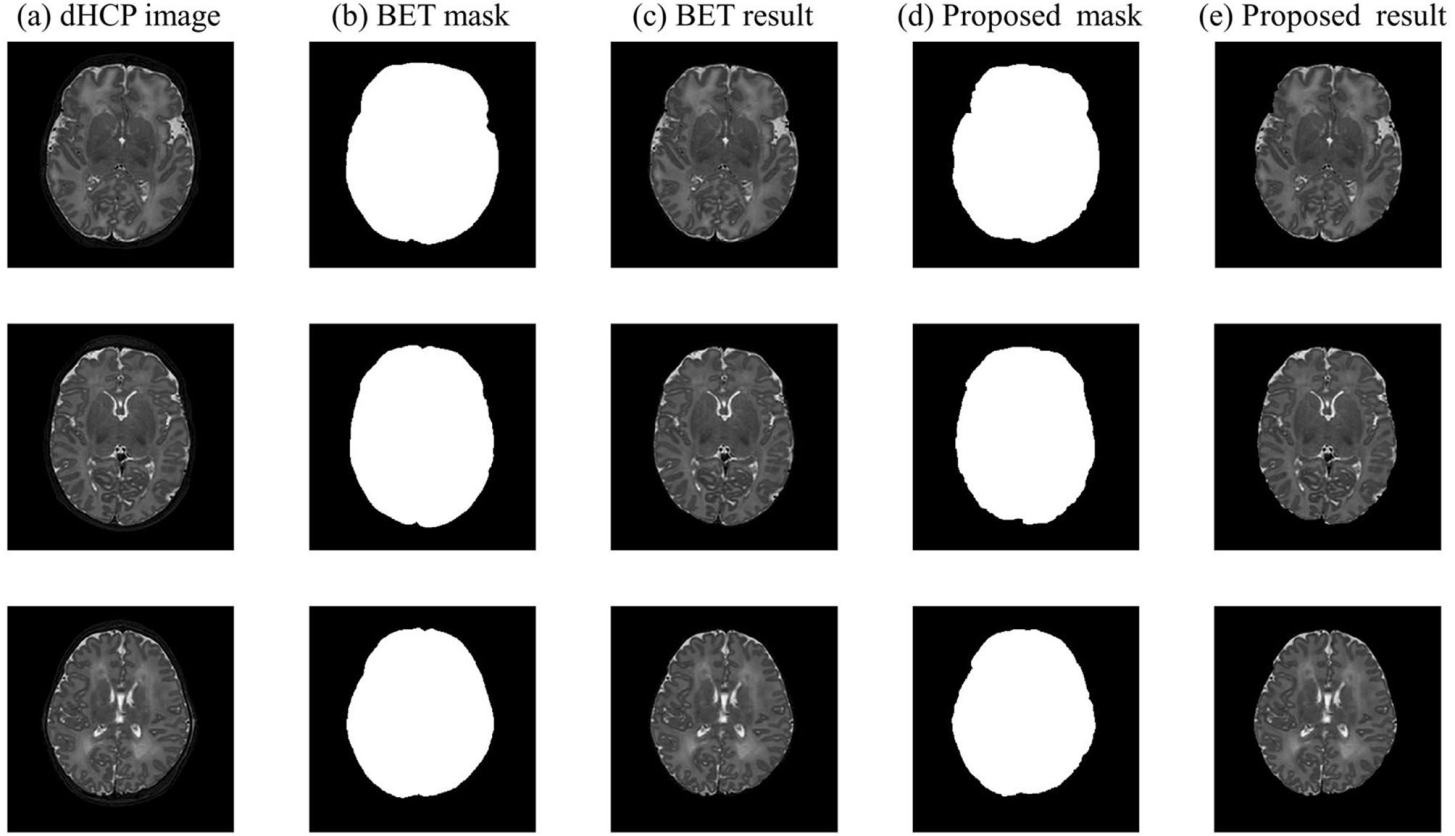
Brain extraction results of BET and the proposed method using the dHCP brain datasets. (**a**) the representative slices of dHCP image, (**b**) BET results provided by dHCP and (**c**) the corresponding brain extraction results, (**d**) masks generated by the proposed method and (**e**) the corresponding brain extraction results.

The quantitative analysis was also performed, as shown in Fig. 7. In Fig. 7a, the Dice coefficient of the proposed method showed the highest values for images acquired with 2D imaging sequences (#1 to #16), followed by iBEAT and BET. The average Dice coefficient was 0.9650 ± 0.006 for the proposed method, 0.9262 ± 0.006 for iBEAT, and 0.9490 ± 0.006 for BET. For the dataset acquired with 3D TSE sequence (#17 to #25), the average Dice coefficient was 0.9746 ± 0.008 for the proposed method, 0.9448 ± 0.004 for iBEAT, and 0.9622 ± 0.01 for BET. As presented in Fig. 8b, the average Precision of 2D imaging sequence dataset was 0.9638 ± 0.009 for the proposed method, 0.8981 ± 0.01 for iBEAT, and 0.9346 ± 0.014 for BET. For data acquired with the 3D imaging sequence, the average Precision was 0.9565 ± 0.016 for the proposed method, 0.8968 ± 0.008 for iBEAT, and 0.9282 ± 0.019 for BET, demonstrating that the proposed method showed higher similarity with manually segmented images and less performance deviations across datasets.

Figure 8 shows selected brain extraction results of the proposed method applied to dHCP dataset and compares them with the brain masks provided by the dHCP (which are basically generated by BET). We have also calculated the average Dice and Precision measures. Since the dHCP datasets was defaced and extraction results around the eyeball areas were generally inaccurate, we calculated the measures for two different cases: for the whole brain slices and for the dataset excluding the slices with the eyeballs. For the whole brain volume, the average Dice coefficients and Precision were 0.9491 ± 0.2750 and 0.9496 ± 0.2830 for the proposed method, and 0.9090 ± 0.2968 and 0.8424 ± 0.2837 for BET, respectively. When the slices with eyeballs were excluded, the average Dice coefficients and Precision increased to 0.9774 ± 0.022 and 0.9638 ± 0.0298 for the proposed method, and 0.9494 ± 0.0415 and 0.9072 ± 0.0655 for BET, respectively. Although the performance of the brain extraction varied from slice to slice (thus larger standard deviation), the results show the feasibility of the proposed method to an external dataset. However, interpretation regarding the experiment with dHCP dataset should be cautiously made as the algorithm was applied to defaced dHCP data.

**Figure 9:**
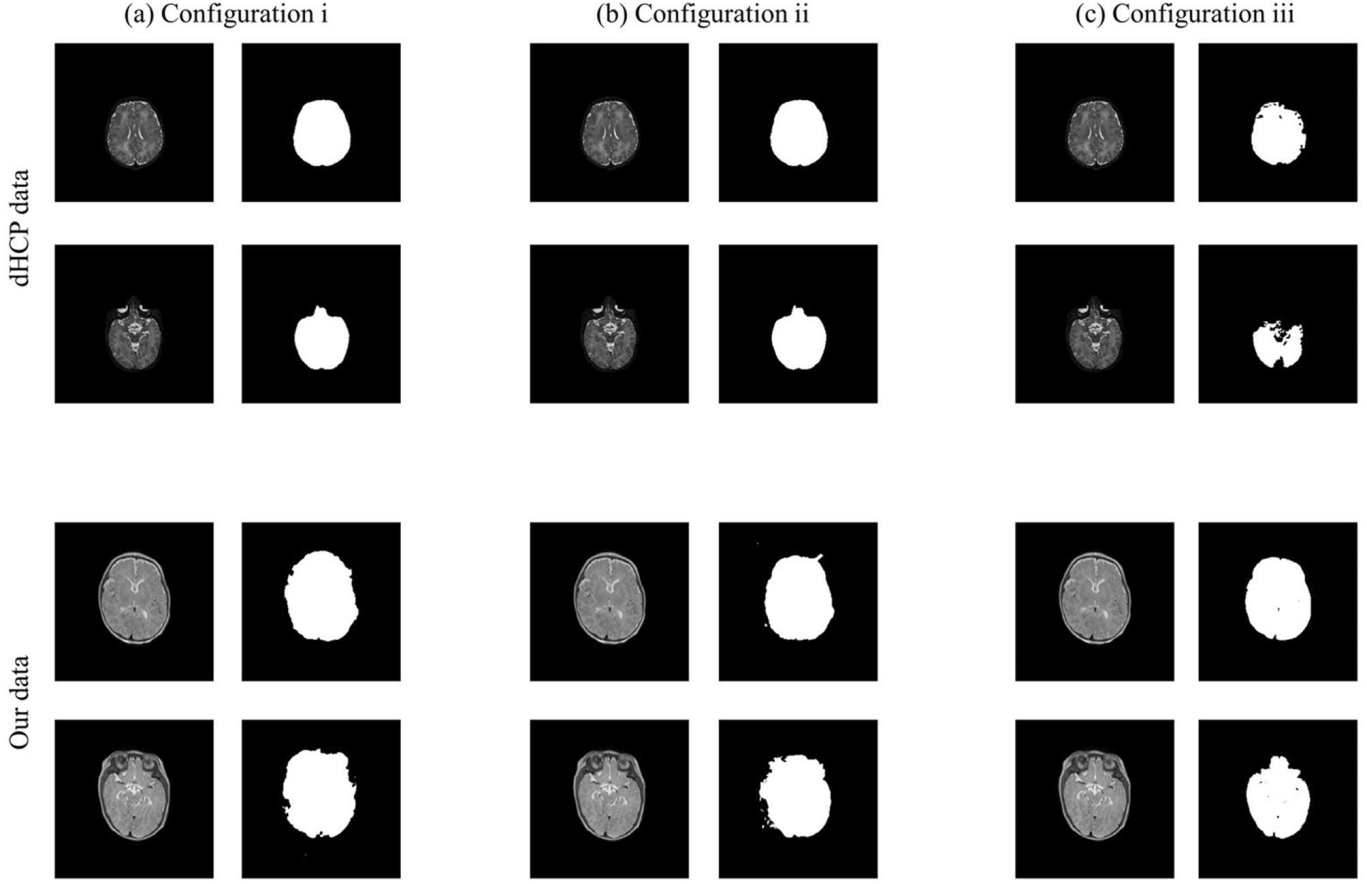
The results of brain extraction using U-net. For training, (**a**) 48 dHCP dataset, (**b**) 44 dHCP + 4 from our dataset, and (**c**) 25 sets from our dataset was used. The brain extraction network trained with each configuration was used to test dHCP and our infant data, as presented in the upper and lower rows, respectively. As demonstrated, the network performs well if the training and test datasets exhibit similar characteristics. When heterogeneous datasets were used for training and testing, the resulting masks tend to be less accurate.

In Fig. 9, brain extraction results generated by U-net are presented. In general, it can be observed that the U-net produced accurate brain masks if the validation set was from the same dataset as the training set (having similar image characteristics), but the masks were less accurate if the training and validation sets exhibited different image characteristics. The quantitative measures were also calculated for the brain masks generated by U-net. When images from the same repository were used for testing and training, the Dice coefficients and Precision were as follows: 0.9606 ± 0.4932 and 0.9350 ± 0.4845 when dHCP data were used as test data for testing configuration i (48 dHCP dataset), 0.9680 ± 0.4925 and 0.9362 ± 0.4848 when dHCP data were used as test data for configuration ii (44 dHCP + 4 from our datasets), and 0.9507 ± 0.4734 and 0.9488 ± 0.4735 when our data were used as test data for configuration iii (25 sets of our data). The quantitative results showed the U-net works well on the similar datasets with training data. However, the Dice coefficients and Precision were slightly lower than those calculated from the brain masks generated by the proposed method.

The general performance of brain extraction was degraded if training and test sets exhibited different characteristics. The average Dice coefficients were 0.5184 ± 0.2688 and 0.6686 ± 0.3581 and the Precision was 0.7419 ± 0.4115 and 0.7748 ± 0.4260 when our dataset was used as test data for configurations i and ii, respectively. The Dice coefficients and Precision were 0.8191 ± 0.4477 and 0.9086 ± 0.5044, respectively, when dHCP dataset was used as test data for the network trained with configuration iii. The results indicate that the characteristics of the training set could result in the performance of brain extraction using U-net.

## Discussion

The brain extraction method for infant T2 weighted brain MR images was proposed based on fuzzy c-means thresholding in this work, with an additional eye removal algorithm. Compared with conventionally used brain extraction algorithms, the proposed method significantly improved the accuracy of skull stripping in infant T2-weighted images. While the conventional algorithms showed errors at the regions with low intensities, the proposed method provided reliable segmentation results at regions with intensity variations. This improvements in skull stripping results can be explained by the use of additional information from neighboring slices. More specifically, the structural information of the previous slice was utilized as additional information to remove the non-brain regions and to prevent elimination of the brain regions with inhomogeneous intensities. The non-brain regions could otherwise be included in the brain mask if the FCM thresholding was exclusively used for brain extraction. In addition, to minimize the errors of including non-brain regions, we also adopted the inverted difference mask during refinement. Furthermore, the features of shape and the location of the eyes were used to remove the eye regions from the brain mask. As conventional brain extraction methods showed difficulties in removing eyes from the brain mask, an additional eye removal procedure was proposed in this work by considering the Hausdorff distance for measuring the shape similarity with a circle-shaped model within the frontal brain regions of high intensities. As we performed the eye removing by evaluating the shape similarity with frontal regions, the proposed method showed improved performance especially in the slices around the eyes.

Based on the experiment results, some observations regarding different brain extraction methods (BET, iBEAT, and U-net) could be made. For example, it was not sufficient to use the BET method for T2-weighted infant brain images although the BET method was generally regarded as an optimal option for brain MR images. While iBEAT and U-net work well for the datasets that have similar characteristics with the training datasets, iBEAT showed overestimated results for the brain mask in our T2-weighted infant datasets, whose image features were different from those of the training datasets. (According to iBEAT, the infant training set included 15 neonates (less than 2 months old), 15 infants (1–3 years old), and 15 children (5–18 years old)). In fact, it is generally the case for machine learning-based algorithms to be optimal for the datasets having similar characteristics with the training datasets. To demonstrate the effect of different training datasets, we have trained the U-net with different combinations of the dHCP dataset and our infant data for brain extraction of infant T2-weighted images. As demonstrated in Results, the tendency of the training datasets can influence the accuracy of the brain extraction. Thus, using a larger amount of data with mixed characteristics may also improve the segmentation performance of the network. However, infant brain images are especially difficult to collect and using a deep-learning network for infant brain segmentation may not be an easy task for such reasons.

Although we have used dHCP dataset as an external validation set and for U-net experiment, it should be mentioned that the publicly available dHCP datasets have been defaced, where the rawdata was also processed for anonymity, especially around the slice with the eyeballs and the face area, and thus the general intensity histogram of the dCHP dataset has been artificially modified. For this reason, user interference was needed during background removal, especially around the slices with eyeballs, to apply the proposed method to the dHCP data and the interpretation of the experiment results regarding dHCP should be carefully made. Nevertheless, the proposed method generated satisfactory brain extraction results for slices without eyes. Again, although dHCP dataset was provided as a modified version, it had to be used as an external validation set for the proposed method because there were no other available options to our knowledge.

Considering the difficulties of acquiring infant brain MR datasets, the proposed algorithm can be more efficiently used for a variety of T2-weighted infant datasets having different characteristics because it utilizes the features of the dataset itself. On the other hand, since a large amount of infant brain images are especially difficult to collect, using a deep-learning network for infant brain segmentation may not be an easy task. However, the proposed method did not show the best performance with regards to the computation time. While other methods (BET, iBEAT, and U-net) take less than 1 min, the proposed method requires 2 to 3 min for processing the whole brain. In addition, the proposed algorithm was not effective for infant T1-weighted images and adult brain images. This may be due to larger variations in image contrasts; the proposed method was optimized for infant T2 weighted MR images, which are known to have less distinct image contrast than adult brain images^11,38^. Nevertheless, the proposed method showed the best Dice and Precision scores in our study, demonstrating that it could be successfully applied for infant T2 weighted brain MR images.

## Conclusion

In an infant brain, the T2-weighted images are often used for research and diagnostic purposes, including the detection of shape changes and brain abnormalities. For these reasons, it is essential to perform brain extraction from the infant T2-weighted brain images. In this study, we proposed an algorithm that can be used to acquire an accurate brain mask from the T2-weighted MR images. The proposed algorithm showed the best performance compared with the conventional algorithms such as BET and iBEAT. We also compared the proposed method with U-net, which is the most frequently used deep-learning network for image processing. The results of the U-net were not successful compared with conventional methods, depending on the size and characteristics of the training datasets. Despite the longer running time, the proposed method can be preferred than the conventional methods, as it provides the highest quantitative measures. This was particularly obvious in the image slices with the eye regions, where the proposed method showed the highest performance while it was challenging for BET and iBEAT to remove the eyes from the brain regions. The proposed method provides a clear brain mask without eye regions. As the proposed method generated more accurate infant brain masks, following processes for brain analysis are expected to provide more reliable features, which can be used as meaningful clues in research and diagnosis.

## Data Availability

The data that support the findings of this study are available from Seoul National University Hospital but restrictions apply to the availability of these data, which were used under license for the current study, and so are not publicly available.
